# Role of tumour markers in monitoring epithelial ovarian cancer

**DOI:** 10.1054/bjoc.2000.1174

**Published:** 2000-04-03

**Authors:** T Meyer, G J S Rustin

**Affiliations:** Department of Medical Oncology, Mount Vernon Hospital, Rickmansworth Road, Northwood, Middlesex, HA6 2RN, UK

## Abstract

Currently the only tumour marker to have a well-defined and validated role in the management of ovarian cancer is CA125. Changes in the level of CA125 can be used as a reliable indication of response or progression according to various criteria, but it does not yet have a clear place in diagnosis or prognosis. Its value as part of a screening tool and during routine follow-up remain the subject of ongoing trials. Other markers remain experimental and do not have a well-defined contribution to make at present. © 2000 Cancer Research Campaign


British Journal of Cancer (2000) **82**(9), 1535–1538
© 2000 Cancer Research Campaign
DOI: 10.1054/ bjoc.2000.1174, available online at http://www.idealibrary.com on IDEAL®

Review

### Role of tumour markers in monitoring epithelial ovarian cancer

**T Meyer and GJS Rustin**

Department of Medical Oncology, Mount Vernon Hospital, Rickmansworth Road, Northwood, Middlesex HA6 2RN, UK

**Summary** Currently the only tumour marker to have a well-defined and validated role in the management of ovarian cancer is CA125. Changes in the level of CA125 can be used as a reliable indication of response or progression according to various criteria, but it does not yet have a clear place in diagnosis or prognosis. Its value as part of a screening tool and during routine follow-up remain the subject of ongoing trials. Other markers remain experimental and do not have a well-defined contribution to make at present. © 2000 Cancer Research Campaign

A wide variety of tumour markers are now available to the clinician caring for patients with malignant disease. They have the potential to contribute to screening, diagnosis and prognosis, as well as providing a means of monitoring response to treatment or indicating relapse during follow-up. Some may also provide specific targets for anti-tumour therapy with antibody-directed treatments or gene therapy. In the majority of cases, however, the role of tumour markers in patient management remains to be fully defined and many markers suffer from poor specificity and/or sensitivity.

In epithelial ovarian carcinoma a number of tumour markers have been identified. The most extensively researched is CA125, a large glycoprotein of unknown function. In addition, several epitopes on the polymorphic epithelial mucin derived from the MUC1 gene have been identified as targets for a family of tumour markers which include CA549, CASA (cancer associated serum antigen), CA19-9, CA15-3, MCA, MOV-1 and TAG72 (Ward, 1994). The cytokeratin proliferation markers TPS and CYFRA21-1 have also been explored in ovarian carcinoma as have enzymes such as placental alkaline phosphatase and biological markers such as CSF and inhibin. In most cases the assays for these markers are not widely available and their relevance to the management of ovarian carcinoma remains to be determined. The exception is CA125 which has been sufficiently well validated to be of use in routine clinical care. For this reason the bulk of the following review will focus on the uses and limitations of CA125 in managing ovarian cancer and the possible contribution of other markers will be discussed where relevant.

### CA125

The CA125 antigen was originally identified following the development of the OC125 antibody, raised by injecting an ovarian cancer cell-line into mice (Bast et al, 1981). The antigen is a 200 000 MW glycoprotein with mainly N-linked glycosylation and is distributed on the endothelium of fallopian tubes, endometrium, endocervix, and also in the normal ovary. In addition, it is found on the mesothelial cells of the pleura, pericardium and peritoneum. The serum concentration of CA125 is elevated by the vascular invasion, tissue destruction and inflammation associated with malignant disease and is elevated in over 90% of those women with advanced ovarian cancer (Tuxen et al, 1995) and 40% of all patients with advanced intra-abdominal malignancy. Levels can also be elevated during menstruation or pregnancy and in other benign conditions such as endometriosis, peritonitis or cirrhosis, particularly with ascites.

*Received 26 November 1999*
*Revised 17 December 1999*
*Accepted 7 January 2000*

*Correspondence to:* GJS Rustin

### Screening

The 5-year survival rate for those with stage I carcinoma of the ovary is around 85% but less than 25% of women present with stage I disease. For the 60% of women that present with stage III or stage IV disease the survival rate drops to less than 20%. Screening, therefore, has the theoretical appeal of improving survival by diagnosing ovarian cancer at a less advanced stage. To date, no randomized trials of ovarian cancer screening have been reported to have a significant impact on the natural history of the disease.

Several studies have examined CA125 as a potential screening tool to assess its feasibility, sensitivity and specificity (Einhorn et al, 1992; Jacobs et al, 1993; 1988; Skates et al, 1995). In a study of 22 000 postmenopausal women, the combination of CA125 with ultrasound achieved a sensitivity for detecting ovarian cancer of 79% at 1-year follow-up. However, the sensitivity of CA125 to detect stage I carcinoma of the ovary is only about 50% (Jacobs et al, 1993) which limits its value as an initial screening tool since picking up stage III disease at an earlier time may not alter outcome. A greater sensitivity for detecting earlier disease has been achieved using a combination of markers and in one study in which CA125, OVX1 and M-CSF were used, 98% of women with stage I ovarian carcinoma had elevated levels of one or more. Unfortunately, specificity is compromised and 11% of healthy women and 51% of women with benign disease also had elevated levels (Woolas et al, 1993). Nevertheless, a large randomized study to assess the feasibility of population screening using CA125 values alone has recently been reported (Jacobs et al, 1999). 21 935 postmenopausal women over the age of 45 were randomized into a control group or a 3-year screening programme. Screening consisted of an annual CA125 measurement which, if elevated above 30 U ml$^{-1}$, was followed by pelvic ultrasound. If, on ultrasound, the volume of the ovary was greater than 8.8 ml, a gynaecological referral was made. The compliance rate was high with 70.7% completing the 3-year screening programme. Of the 10 958 women entering the screening arm 468 (4.3%) had a raised CA125, 29 (0.26%) had surgical investigation and six (0.055%) had screen-detected ovarian cancer of which three had potentially curable stage I or II disease (Jacobs et al, 1999). The positive predictive value of the screening strategy was 21% but the trial was insufficiently large to detect significant differences in mortality.

Further studies with larger numbers of women must be undertaken to establish if there is any real benefit in terms of survival and to address the psychosocial and health economic issues raised by such a programme. Apart from women at high risk, who may be eligible for the ongoing UKCCR trial, ovarian cancer screening with CA125 is not currently recommended (Department of Health, 1999).

### Diagnosis

No single marker has been shown to be sufficiently sensitive or specific to contribute to the diagnosis of ovarian cancer. For example, CA125 levels above 35 U ml$^{-1}$ will be found in 1% of the normal population, 6% of patients with benign disease, 28% of individuals with non-gynaecological malignancy and 82% of patients with epithelial ovarian cancer (Bast et al, 1983). The level of CA125 taken in conjunction with pelvic ultrasound and menopausal status may help further to distinguish benign from malignant disease (Jacobs et al, 1990). Combinations of markers may be of value and a sensitivity of 73% and specificity of 100% has recently been reported using the combination of CA125 and D-dimer analysis in a series of 56 women with epithelial ovarian cancer and 65 with benign ovarian disease (Gadducci et al, 1996). In another study the combination of five different tumour markers (CA125, OVX1, LASA, CA15-3 and CA72-4) achieved a specificity of 93% and a sensitivity of 90.6% in differentiating benign from malignant disease using logistic regression analysis (Woolas et al, 1995). In practice, however, the diagnosis will follow surgery and histological evaluation in the majority of cases, and tumour markers are unlikely to contribute.

### During initial management

Levels of CA125 may be elevated by tissue damage for 2 weeks following surgery and residual disease should be suspected if levels plateau above the upper limit of normal or rise after this interval. With a half-life of 6 days, CA125 may take many weeks to return to normal after surgery. Thus, unless a pre-chemotherapy level is the same or higher than a previous sample taken after surgery, any subsequent fall would be due to surgery and/or chemotherapy. Conversely, a rising CA125 level may indicate progressive disease in the 20% of women who do progress during their initial treatment. Earlier detection of progressive disease could spare the patient further ineffective treatment and considerable savings can be made in terms of therapy and investigations (Rustin et al, 1992). Criteria for progression need to be defined if it is to be relied upon as a single indicator of treatment failure. Such analysis must be highly specific in order that effective treatment is not stopped prematurely and any consequent loss of sensitivity means that progressing patients would be picked up by conventional clinical means rather than CA125. Using a rise of 25% in the CA125, confirmed by a third sample, has been shown to yield a specificity of 100% and a sensitivity of 40% (Rustin et al, 1992).

### During routine follow-up

In about 70% of patients a rising CA125 may be the first indication of relapse, predating clinical relapse by a median of 4 months (Rustin et al, 1996*b*; van der Burg et al, 1990). Furthermore, since performance status, tumour bulk and number of tumour lesions are independent prognostic factors for response, progression-free and overall survival, one might speculate that earlier detection of relapse would be beneficial (Eisenhauer et al, 1997). However, the value of routinely measuring CA125 during the follow-up of women after initial treatment is uncertain and practice varies. Some clinicians routinely perform CA125 measurements and treat asymptomatic patients on the basis of a rising level alone, while others ignore a rising level until symptoms warrant treatment or do not measure CA125 at all. It must be remembered that relapse after initial treatment is usually incurable and any theoretical advantage of instituting palliative chemotherapy at an earlier stage must be weighed against the anxiety, regular venepuncture and loss of treatment-free time which arises as a consequence of such monitoring. The impact of such monitoring on overall survival, symptom-free survival and quality of life is currently being assessed in a multicentre MRC/EORTC trail in which patients who are in remission following initial treatment are randomized between immediate treatment (within 4 weeks) of relapse by CA125 criteria or treatment only on clinical relapse.

Until the results of this trial are known, CA125 should not be routinely monitored and its measurement should be reserved as an aid to the diagnosis of relapse when clinically suspected.

### Defining relapse during follow-up by CA125

If CA125 is to be relied upon as an indicator of relapse in the absence of disease evaluable by other methods, it must be sufficiently sensitive to reliably confirm clinically-suspected relapse or demonstrate progressive disease on treatment. Moreover, it should be highly specific in order that effective therapy is not stopped prematurely on account of a false-positive result.

A rise of 50% (Krebs et al, 1986) or 100% (Bast et al, 1983) has been shown to be predictive of progression and in one study a level of CA125 above the normal range was found in 73% of relapsed patients (van der Burg et al, 1990). In this study the addition of physical and gynaecological examination increased the detection of relapse up to 92%, so that the addition of radiological investigations or surgery only contributed to the diagnosis in 8% of cases (van der Burg et al, 1990).

Several studies have analysed definitions of progression according to CA125. In one such study of 255 patients following initial chemotherapy, a doubling of CA125 above the upper limit of normal was found to have a sensitivity of 85.9%, a specificity of 91.3% and a positive predictive value of 94.8% for indicating progression. The addition of a confirmatory raised level reduced the false-positive rate to 2% and progression by these criteria provided a median lead time of 63 days over standard clinical criteria (Rustin et al, 1996*b*). This definition has been adopted for the MRC/EORTC CA125 follow-up trial discussed above. Clearly these data suggest that CA125 is able to reliably indicate relapse

 *© 2000 Cancer Research Campaign*

and, when accompanied by signs or symptoms compatible with relapse, is frequently used to instigate further chemotherapy.

### The use of CA125 as a marker of response to therapy

Many patients with advanced ovarian carcinoma are ineligible for clinical trials because their disease is not evaluable according to the standard response criteria as defined by the World Health Organisation (WHO), the Gynaecology Oncology Group (GOG) or the Eastern Cooperative Oncology Group (ECOG) (Blessing, 1990; Miller et al, 1981; Oken et al, 1982). Defining response according to CA125, which is elevated in 95% of women with advanced disease, therefore presents the possibility of entering more women into clinical trails, as well as obviating the need for intensive radiological investigation. Several definitions have been proposed (Markman, 1993; Ng et al, 1989; Rustin et al, 1993) but the only definition that has been validated prospectively is that of Rustin (Rustin et al, 1996*a*). In this study, CA125 response criteria were generated by examining data from 277 patients participating in the North Thames Ovary trial of maintenance radiotherapy versus carboplatin. A biological response was scored for an individual patient if there was either a 50% or 75% decrease in CA125 levels calculated according to mathematical logic by a computer program, to avoid mistakes and to provide the precision required by clinical trials (Rustin et al, 1996*a*; Rustin et al, 1999). A 50% response was defined as a 50% decrease in the CA125 level following two levels which were elevated, and this decrease was confirmed by a fourth sample. A 75% response occurred if there was a fall in the value of the CA125 by 75% over three samples. In each case the final sample had to be analysed at least 28 days from the preceding sample. These response definitions were then tested in two other groups of patients; 254 from the North Thames Ovary five versus eight trial and 458 from the GOG dose-intense versus standard chemotherapy trail. Of the 620 patients that were assessable for response according to CA125 only two (0.3%) had a CA125 response at the time of clinical progression. In the GOG trial, CA125 response was 66% in all 317 patients assessable for CA125 and 67% in 221 patients who were not measurable according to GOG criteria. This compares with a GOG-defined response rate of 62%. Thus using these criteria CA125 can be used to accurately determine response rates to first-line chemotherapy.

Subsequently, these CA125 response definitions have been validated in a large number of clinical trials including drugs such as cisplatin and paclitaxel (Bridgewater et al, 1999). A recent analysis of 1396 patients involved in the testing of 14 investigational drugs for relapsed ovarian cancer in phase II clinical trials found that CA125 response rates concurred with standard response rates and could therefore provide a reliable, cheaper and more available means of identifying active drugs worthy of further study (Rustin et al, 2000).

For managing the individual patient many factors are considered when deciding whether or not to continue with therapy and the rise or fall of the CA125 can make a useful contribution. Caution is needed since levels in the individual undergo random fluctuation and can fall in response to the drainage of malignant effusions or ascites. Some authors have raised concerns that specific drugs such as paclitaxel may render CA125 levels unreliable (Davelaar et al, 1996; Pearl et al, 1994) for indicating response. This question has recently been re-examined in 144 patients treated with paclitaxel in four different trials using the 50% and 75% response criteria defined above (Bridgewater et al, 1999). The progression-free survival for responders compared with non-responders was equivalent regardless of whether CA125 or standard response criteria were used. Furthermore, the false-positive rate for a CA125 response was less than 3% suggesting that, if the CA125 suggests a response, then there is a response in 97% of cases and radiological reassessment is probably not warranted. False-negative rates, however, were higher at about 21% and stopping treatment on the basis of absence of response according to CA125 alone would therefore risk under-treating patients. Such under-treatment could be avoided if therapy were continued until there was evidence of progression by clinical, radiological or CA125 criteria (Rustin et al, 1999).

### Prognosis

The value of tumour markers as indicators of prognosis has been explored both at diagnosis and during initial treatment.

Studies examining the prognostic implications of preoperative CA125 have been contradictory (Tuxen et al, 1995) and many investigators have found CA125 to be of no prognostic value (Sevelda et al, 1989; van der Burg et al, 1988). However, in one study of 201 patients with stage I disease, CA125 emerged as a powerful prognostic factor for survival by multivariate analysis (Nagele et al, 1995). Those women with a CA125 above 65 U $ml^{-1}$ had a 6.37-fold risk of dying from disease compared to those with levels below this cut-off. With current practice of giving adjuvant chemotherapy for stage IC disease and above, the identification of other high-risk stage I patients may be of clinical value. Other markers may emerge as of greater prognostic significance than CA125 and in one recent preliminary report, CA125 was compared with serum levels of the soluble urokinase-type plasminogen activator receptor (suPAR). While CA125 was found to be a more specific marker of ovarian cancer than suPAR, high preoperative levels of suPAR were associated with a worse survival, whereas CA125 was found to have no prognostic significance (Seir et al, 1998).

Several studies have examined CA125 as a prognostic tool during initial chemotherapy and again its use in this setting is limited. Initial small studies suggested that the half-life (van der Burg et al, 1988) or regression parameter for CA125 (Buller et al, 1992) in an individual could be used to divide patients into better or worse prognostic groups, while in other studies the division was made using an optimum cut-off for the CA125 value after one, two or three courses of chemotherapy (Lavin et al, 1987; Sevelda et al, 1989). A larger study subsequently re-evaluated several of these methods in a patient group of 248 and found that the best predictive measurement was the value of the CA125 prior to the third course of treatment (Fayers et al, 1993). Using a cut-off of 70 IU $ml^{-1}$, 57% with a level above this were correctly predicted to progress or die within 12 months while 80% of those with a level below 70 IU $ml^{-1}$ were alive and progression-free. However, this still means that 43% patients with a CA125 above 70 after two courses of treatment were alive and well at 12 months. A decision to abandon further treatment at that stage or give more aggressive treatment may well have been misguided. The authors consequently conclude that the prognostic information gained from CA125 alone is not sufficiently accurate to manage individual patients during initial chemotherapy.

This view is supported by the work of Peters-Engl et al reported in this journal. In this study a regression parameter was defined

*© 2000 Cancer Research Campaign* 

using the CA125 level obtained preoperatively and that at 3 months postoperatively after two cycles of chemotherapy. This parameter proved to be a significant prognostic factor during the first 12 months, but thereafter it failed to discriminate between long- or short-term survivors. Nevertheless, although CA125 may be a poor predictor of long-term prognosis, it is accurate at indicating actual tumour progression. Again, there has been some interest in whether combinations of tumour markers may be of value in improving the detection of residual disease during initial treatment. Elevated OVX1 levels have been found in 70% of patients with carcinoma of the ovary but only 5% of the normal population. When used in combination with CA125 in patients undergoing second-look surgery, 56% of women were correctly identified as having residual disease compared with 35% using CA 125 alone (Xu et al, 1993).

### REFERENCES

Bast RC, Feeny M, Lazarus H, Nadler LM, Colvin RB and Knapp RC (1981) Reactivity of a monoclonal antibody with human ovarian carcinoma. *J Clin Invest* **68**: 1331–1337

Bast RC, Klug TL, John ES and Al E (1983) A radioimmunoassay using a monoclonal antibody to monitor the course of epithelial ovarian cancer. *N Engl J Med* **309**: 883–887

Blessing JA (1990) Design, analysis, and interpretation of chemotherapy trials in gynecologic cancer. In: Deppe G (ed) *Chemotherapy of Gynecologic Cancer,* 2nd edn. Wiley-Liss: New York, pp. 63–97

Bridgewater JA, Nelstrop AE, Rustin GJS, Gore ME, McGuire WP and Hoskins WJ (1999) Comparison of standard and CA125 response criteria in patients with epithelial ovarian cancer treated with platinum or paclitaxel. *J Clin Oncol* **17**: 501–508

Buller RE, Berman ML, Bloss JD, Manetta A and DiSaia PJ (1992) Serum CA125 regression in epithelial ovarian cancer: correlation with reassessment findings and survival. *Gynecologic Oncology* **47**: 87–92

Davelaar EM, Bonfrer JMG, Verstraeten RA, ten Bokkel Huinink WW and Kenenmans P (1996) CA125 A valid marker in ovarian carcinoma patients treated with paclitaxel. *Cancer* **78**: 118–127

Department of Health (1999) Improving outcomes in gynaecological cancers. DoH: London

Einhorn N, Sjovall K, Knapp RC, Hall P, Scully RE, Bast RC and Zurawski VR (1992) Prospective evaluation of serum CA125 levels for early detection of ovarian cancer. *Obstet Gynecol* **80**: 14–18

Eisenhauer EA, Vermorken JB and van Glabbeke M (1997) Predictors of response to subsequent chemotherapy in platinum pretreated ovarian cancer: A multivariate analysis of 704 patients. *Ann Oncol* **8**: 963–968

Fayers PM, Rustin G, Wood R, Nelstrop A, Leonard RCF, Wilkinson P, Cruickshank D, McAllister EJ, Redman CWE, Parker D, Scott IV, Slevin ML and Roulston JE (1993) The prognostic value of serum CA125 in patients with advanced ovarian carcinoma: an analysis of 573 patients by the Medical Research Council Working Party on Gynaecological Cancer. *Int J Gynecol Cancer* **3**: 285–292

Gadducci A, Baichhi U, Marrai R, Ferdegini M, Bianchi R and Facchini V (1996) Preoperative evaluation of D-dimer and CA125 levels in differentiating benign from malignant ovarian masses. *Gynecologic Oncology* **60**: 197–202

Jacobs I, Stabile I, Bridges J, Kemsley P, Reynolds C, Grudzinskas J and Oram D (1988) Multimodal approach to screening for ovarian cancer. *Lancet* 268–271

Jacobs I, Oram D, Fairbanks J, Turner J, Frost C and Grudzinskas J (1990) A risk of malignancy index incorporating CA125, ultrasound and menopausal status for the accurate preoperative diagnosis of ovarian cancer. *Br J Obstet Gynaecol* **97**: 922–929

Jacobs I, Davies AP, Bridges J, Stabile I, Fay T, Lower A, Grudzinkas JG and Oram D (1993) Prevalence screening for ovarian cancer in postmenopausal women by CA125 measurement and ultrasonography. *BMJ* **306**: 1030–1032

Jacobs IJ, Skates SJ, MacDonald N, Menon U, Rosenthal AN, Davies AP, Woolas R, Jeyarajah AJ, Sibley K, Lowe DG and Oram DH (1999) Screening for ovarian cancer: a pilot randomised study. *Lancet* **353**: 1207–1210

Krebs H, Goplerud DR, Kilpatrick SJ, Myers MB and Hunt A (1986) Role of CA125 as tumour marker in ovarian carcinoma. *Obstet Gynecol* **67**: 473–477

Lavin PT, Knapp RC, Malkasian G, Whitney CW, Berek JC and Bast RC (1987) CA125 for the monitoring of ovarian carcinoma during primary therapy. *Obstet Gynecol* **69**: 223–227

Markman M (1993) A proposal to use CA125 to evaluate activity of new antineoplastic agents in ovarian cancer. *Gynecologic Oncology* **51**: 297–298

Miller AB, Hoogstraten B, Staquet M and Winkler A (1981) Reporting results of cancer treatment. *Cancer* **47**: 207–214

Nagele F, Petru E, Medl M, Kainz C, Graf AH and Sevelda P (1995) Preoperative CA125: an independent prognostic factor in patients with Stage I epithelial ovarian cancer. *Obstet Gynecol* **86**: 259–264

Ng LW, Homesley HD, Barrett RJ, Welander CE and Case LD (1989) CA125 values predictive of clinical response during second-line chemotherapy for epithelial ovarian cancer. *Am J Clin Oncol* **12**: 106–109

Oken MM, Creech RH, Tormey DC, Horton J, Davis TE, McFadden ET and Carbone PP (1982) Toxicity and response criteria of the Eastern Cooperative Oncology Group. *Am J Clin Oncol* **5**: 649–655

Pearl ML, Yashar CM, Johnstone CM, Reynolds RK and Roberts MD (1994) Exponential regression of CA125 during salvage treatment of ovarian cancer with Taxol. *Gynecologic Oncology* **53**: 339–343

Rustin GJ, Nelstrop A, Stilwell J and Lambert HE (1992) Savings obtained by CA125 measurements during therapy for ovarian carcinoma. The North Thames Ovary Group. *Eur J Cancer* **28**: 79–82

Rustin GJ, van der Burg ME and Berek JS (1993) Advanced ovarian cancer. Tumour markers. *Ann Oncol* **4** (Suppl 4): 71–7

Rustin GJ, Nelstrop AE, McClean P, Brady MF, McGuire WP, Hoskins WJ, Mitchell H and Lambert HE (1996*a*) Defining response of ovarian carcinoma to initial chemotherapy according to serum CA125. *J Clin Oncol* **14**: 1545–1551

Rustin GJ, Nelstrop AE, Tuxen MK and Lambert HE (1996*b*) Defining progression of ovarian carcinoma during follow-up according to CA125: a North Thames Ovary Group Study. *Ann Oncol* **7**: 361–36

Rustin GJS, Nelstrop AE, Bentzen SM, Piccart MJ and Bertelsen K (1999) Use of tumour markers in monitoring the course of ovarian cancer. *Ann Oncol* **10**: S21–S27

Rustin G, Nelstrop A, Bentzen S, Bond S and McClean P (2000) Selection of active drugs for ovarian carcinoma based on CA125 and standard response rates in phase II trials. *J Clin Oncol* (in press)

Seir CF, Stephens R, Bizik J, Mariani A, Bassan M, Pedersen N, Frigerio L, Ferrari A, Dano K, Brunner N and Blasi F (1998) The level of urokinase-type plasminogen activator receptor is increased in serum of ovarian cancer patients. *Cancer Res* **58**: 1843–1849

Sevelda P, Schemper M and Spona J (1989) CA125 as an independent prognostic factor for survival in patients with epithelial ovarian cancer. *Am J Obstet Gynecol* **161**: 1213–1216

Skates SJ, Xu F, Yu Y, Sjovall K, Einhorn N, Chang Y, Bast RC and Knapp RC (1995) Toward an optimal algorithm for ovarian cancer screening with longitudinal tumour markers. *Cancer* **76**: 2004–2010

Tuxen MK, Soletormos G and Dombernowsky P (1995) Tumour markers in the management of patients with ovarian cancer. *Cancer Treat Rev* **21** 215–245

van der Burg MEL, Lammes FB, van Putten WLJ and Stoter G (1988) Ovarian cancer: the prognostic value of the serum half-life of CA125 during induction chemotherapy. *Gynecologic Oncology* **30**: 307–312

van der Burg MEL, Lammes FB and Verweij J (1990) The role of CA125 in the early diagnosis of progressive disease in ovarian cancer. *Ann Oncol* **1**: 301–302

Ward BW (1994) Tumour markers in ovarian cancer: the current state of clinical use. *Klinisches Labor* **40**: 1227–1231

Woolas RP, Xu FJ, Jacobs IJ, YH Y, Daly L, Berchuck A, Soper JT, Clarke-Pearson DL, Oram DH and Bast RCJ (1993) Elevation of multiple serum markers in patients with stage I ovarian cancer. *J Natl Cancer Inst* **85**: 1748–1751

Woolas RP, Conaway MR, Xu F, Jacobs IJ, Yu Y, Daly L, Davies AP, O'Briant K, Berchuck A and Soper JT (1995) Combinations of multiple serum markers are superior to individual assays for discriminating malignant from benign pelvic masses. *Gynecologic Oncology* **59**: 111–116

Xu F-J, Yu Y-H, Daly L, DeSombre K, Anselmino L, Hass GH, Berchuck A, Soper JT, Clarke-Pearson DL, Boyer C, Layfield LJ and Bast RCJ (1993) OVX1 radioimmunoassa complements CA125 for predicting the presence of residual ovarian carcinoma at second look surgical surveillance procedures. *J Clin Oncol* **11**: 1506–1510

*© 2000 Cancer Research Campaign*
